# Innovations and adaptations in neonatal and pediatric respiratory care for resource constrained settings

**DOI:** 10.3389/fped.2022.954975

**Published:** 2022-10-31

**Authors:** Andrew Wu, Mariya Mukhtar-Yola, Sreyleak Luch, Stephen John, Bikash Raj Adhikari, Caitlin Bakker, Tina Slusher, Ashley Bjorklund, Jameel Winter, Chinyere Ezeaka

**Affiliations:** ^1^ Division of Critical Care Medicine, Department of Anesthesiology, Critical Care and Pain Medicine, Boston Children’s Hospital, Boston, MA, United States; ^2^ Department of Paediatrics, National Hospital Abuja, Abuja, Nigeria; ^3^ Department of Pediatrics, Chenla Children’s Healthcare, Kratie, Cambodia; ^4^ Department of Pediatrics, University of Minnesota Medical School, Minneapolis, MN, United States; ^5^ Department of Pediatrics, United Mission Hospital Tansen, Tansen, Palpa, Nepal; ^6^ Discovery Technologies, Health Sciences Libraries, University of Minnesota, Minneapolis, MN, United States; ^7^ Department of Pediatrics, Hennepin Healthcare, Minneapolis, MN, United States; ^8^ Department of Paediatrics, College of Medicine, University of Lagos, Lagos, Nigeria

**Keywords:** global health, neonatology, respiratory support, innovations, medical devices, bubble CPAP

## Abstract

Respiratory disease is a leading cause of death in children under 5 years of age worldwide, and most of these deaths occur in low- to middle-income countries (LMICs) where advanced respiratory care technology is often limited. Much of the equipment required to provide advanced respiratory care is unavailable in these areas due to high costs, the need for specialty trained personnel, and myriad other resource constraints that limit uptake and sustainable use of these devices, including reliable access to electricity, sensitive equipment needing frequent maintenance, single-patient-use supplies, and lack of access to sterilization equipment. Compounding the problem, pediatrics is uniquely challenging in that one size does not fit all, or even most patients. Despite these substantial barriers, numerous innovations in respiratory care technology have been made in recent years that have brought increasing access to high quality respiratory care in some of the most remote areas of the world. In this article, we intend to review the global burden of respiratory diseases for children, highlight the prototypical innovations that have been made in bringing respiratory care to LMICs, spotlight some of the technologies being actively developed to improve respiratory care in resource-constrained settings, and conclude with a discussion highlighting areas where further innovation is still needed.

## Introduction

Some of the leading causes of pediatric morbidity and mortality worldwide such as lower respiratory tract infections (LRTIs) and prematurity require advanced respiratory support as central components of their management. LRTIs are the leading cause of death in children under five years of age worldwide (1). Furthermore, nearly 45% of all under-5 deaths occur in the neonatal period, with infections, birth complications and prematurity accounting for over 80% of these deaths (2). A disproportionate number of these deaths–approximately 80%–occur in low- to middle-income countries (LMICs) where the resources, skills, and technology required to adequately care for children being treated for LRTIs are often lacking (3). There are numerous reasons for this resource scarcity including high monetary costs, specialized training for personnel, unreliable access to electricity, and lack of sterilization (4). Therefore, a need exists to provide cost-conscious technologies and techniques that can effectively provide respiratory support for this vulnerable population.

Costs of respiratory support technology are high and not often realized by providers who primarily work at the bedside. A standard ventilator unit usually costs between $30,000 and $50,000 USD (5), while a typical continuous positive airway pressure (CPAP) or bi-level positive airway pressure (BiPAP) device typically costs between $1,000 and $10,000 USD (6). Oxygen blenders and flow meters often cost around $1,000 USD, as do some humidifiers (7) and these costs are beyond the budget for most health facilities. In 2019, the mean health expenditure of low income and low middle income countries was 5.4% of gross domestic product (GDP), in comparison to high income and upper middle-income countries, which had a mean expenditure of 7.2% of GDP. Given that the average GDP of a high-income country (HIC) was over 5.9 times that of an LMIC, this gap is even more profound (8). The spending burden is further compounded by the necessary purchase of multiple devices in addition to the cost of cleaning, maintenance, repairs and supplies. Other costs and barriers to operationalizing innovations in any low-resource setting (LRS) include the cost of time, including the time to build a device and/or time to deliver a product which can also impact how quickly the innovation can be made available, and labor costs, including any specially trained personnel for appropriate maintenance and repairs; this can impact the sustainability of a given technology and subsequently affect the overall cost-effectiveness of the innovation (9).

Additional barriers to providing adequate respiratory support in LMICs include but are not limited to dependence on electricity when reliable access is not guaranteed; limited supplies of oxygen and/or compressed air; limited tools or spare parts required for maintenance or repair; lack of access to sterilization techniques and technology; proprietary parts to equipment which limits options for replacement materials; and single use devices which ultimately add cost and increase medical waste (10). Therefore, innovations that do not rely on electricity and minimize utilization of consumables like oxygen can be beneficial. Devices that are reusable, low-cost, rugged, use materials with high durability, and/or operate through relatively simple and understandable mechanics are preferable.

Compounding these issues are unique challenges such as greater variability in size and physiology associated with caring for children. This significantly impacts the design of any given device and may influence the appropriate size of a breathing interface or the minimum respiratory support that the device can deliver (11). There are also specific clinical and physiologic targets that are different depending on age and disease process, to which the device should ideally be able to adapt. For example, the precision with which tidal volumes can be altered should ideally be greater for smaller children since small changes in volumes can have a greater impact in this population (12). Designing therapies intended to treat common pediatric conditions such as respiratory distress syndrome, pneumonia, asthma, and bronchiolitis should take these factors into account.

Once a device has been prototyped, it needs to be validated in a relevant clinical setting before it can be used confidently; however, designing and conducting such studies in LRS can be difficult. Monetary and time costs to research already exist as barriers in HICs and can be even more difficult to overcome in a LRS (13, 14). Uniquely, for any international exchange, the complexity of travel logistics is often part of the planning process which can further complicate the validation process. Once implementation occurs, ongoing training is a necessary step to optimizing sustainability.

Finally, the technology sector has historically been motivated by intellectual property, marketability, and revenue, which is not often central to the motivation behind low-cost device development and frugal innovation (15). Global health work is often collaborative in nature and aims to find mutually beneficial ground for all parties. In this way, innovations are ideally generated out of and exist within a partnership that is also mutually beneficial, as opposed to the more competitive spirit that exists in traditional technology development and industry. As an example, open-source devices represent a field of innovation that focuses more on providing a service to a large audience than owning particular property or benefiting from profit (16).

Given all these barriers, it comes as no surprise that development, implementation, and maintenance of innovations to support child health in LRS have made slow progress. Nonetheless, progress has been made, and in this review, we will highlight several innovations that aim to lessen the burden of respiratory diseases in children living in LRS.

## Innovations in respiratory care for LMICs

For new medical devices to be successfully implemented in LMICs, they need to satisfy certain criteria (Table 1). The prototypical example of a successful adaptation of medical technology, and one that has a substantial body of evidence to support its efficacy, is the home-made spacer for metered dose inhalers (17), which can be easily constructed from a disposable plastic water bottle, and modified to be used as a face-mask, or mouth-piece interface. This simple intervention is implementable around the world at almost no cost. Most medical devices developed for LMICs will not be quite as cost-effective as this example. However, this example is worth mentioning because adaptation can be a powerful tool. In this section, we will highlight some of the ongoing innovations being developed and implemented around the world to improve the care of children with respiratory illnesses. It is important to note, that the many of the highlighted innovations are from our group of authors. There are many other innovations that are being developed globally that are not described in detail here but have similar concepts and aims of improved access to care. Examples of these include other CPAP device such the Pumani CPAP device which was described in the 2013 article by Brown et al. and the Diamedica baby CPAP device as well as low cost ventilator such as the MADVent developed for patients with COVID-19 and the noninvasive pressure support ventilator described by Garmendia et al. in their 2020 article (18–21).

**Table 1:** Criteria for medical equipment for resource limited settings.

**Criteria for Medical Equipment for Resource Constrained Settings**
Affordable
Not dependent on continuous electricity
Easy to set up, monitor and troubleshoot
Spare parts available
Easy to disassemble and clean
Optimized for pediatric patients
Robust design without sensitive materials that can break

### Low-cost bubble CPAP

Devices that support breathing through the delivery of CPAP have long been recognized as important therapeutic interventions decreasing mortality from respiratory illness in all age groups (22–24). CPAP helps maintain lung volumes during exhalation, improves oxygenation, and decreases respiratory muscle fatigue. In children with respiratory distress, CPAP has become a standard intervention utilized to attempt stabilization prior to advancing to invasive mechanical ventilation and has decreased mortality in high-income countries (24–26). One means of delivering CPAP, ventilator-derived CPAP, is costly, relies on a reliable source of electricity, and requires intensive expert monitoring and advanced biomedical support, some or all of which are often lacking in hospitals in LMICs (27).

**Figure 1:**
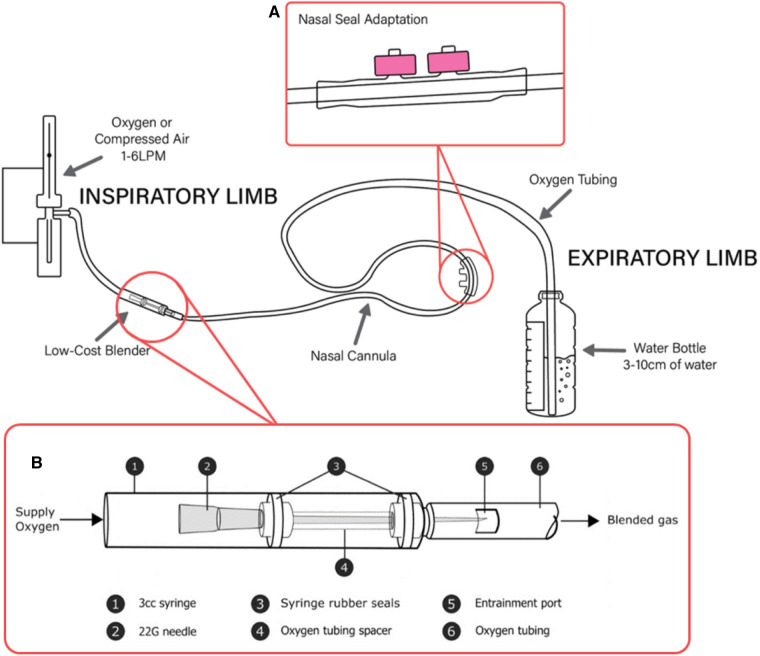
A low-cost bCPAP circuit with SEAL-bCPAP modification and low-cost blender modification. (**A**) SEAL-bCPAP Modification. (**B**) Low-cost Oxygen Blender Graphic credit: Mara T. Halvorson.

“Bubble CPAP” (bCPAP) is another type of CPAP that has been successfully implemented in low and high resource settings. The bCPAP circuit generates CPAP by submerging the distal aperture of the expiratory limb under water (Figure 1). The depth of the tube in the water determines the positive end expiratory pressure that is generated within the tubing. The water bubbles as exhaled air escapes against the pressure (28). Commercial bCPAP devices are still very costly ($3,000–$6,000 USD) (29); however, less expensive versions such as the Pumani and Vayu devices have been designed (18, 30) and simplified versions, as published by the WHO, can be constructed even more inexpensively ($4–5 USD) with easy to obtain supplies (31). Some of these simplified versions can function with compressed air and/or cylinder supplied oxygen and therefore do not require electricity. Because they use a nasal prong or mask interface, less intensive monitoring is required than with mechanical ventilation (32, 33).

There is a growing body of literature supporting bCPAP use, including low-cost versions, in neonatal respiratory distress in both high and low-income settings (28, 33, 34). A study in 2007 showed a 33% reduction in mortality using commercial bCPAP compared to conventional CPAP in premature infants (35). In 2014, a systematic review of 14 studies observing commercial bCPAP use in neonates in LMICs showed a reduction in the need for mechanical ventilation by 30%–50% when using bCPAP compared to oxygen therapy. The analysis also found a lower clinical failure rate when using bCPAP compared to conventional CPAP in low-resource settings (36). Reported complications associated with bCPAP are similar to those seen with all forms of non-invasive ventilation, including nasal tissue irritation (10%–13%) (35, 37) and aerophagia resulting in gastric distension (5%–15%) (38), with less frequently reported serious complications of aspiration (<1%), nasal septal necrosis (<1%), and pneumothorax (<1%) (36, 39). Overall, bCPAP is considered a safe treatment option in neonates and is endorsed by the World Health Organization (WHO) for this purpose (31).

While the evidence that bCPAP is effective in neonates is clear, much less is known about its use in older infants and children. The efficacy, safety, and feasibility of bCPAP for use in children beyond the neonatal age group has yet to be conclusively demonstrated. High-income countries use ventilator-derived CPAP in older children. In LRSs, there have been five small observational clinical studies evaluating bCPAP use in older infants and children (22, 40–43). These studies provide preliminary evidence that bCPAP appears to be safe and feasible, but efficacy has not been conclusively demonstrated possibly due to issues with nasal seal and leak in older children. A low-cost (∼$5 USD) modified bCPAP circuit (Simplified Earplug Adapted-bCPAP, “SEAL-bCPAP,” (Figure 1A), has been developed and tested for safety for use in children (32). SEAL-bCPAP is constructed with easy-to-obtain and inexpensive materials. The addition of commercial earplug material around the nasal prong produces an improved fit at the nasal interface to decrease leak by creating a soft seal. This modification has been shown to be safe, and there was a trend toward improved efficacy, but further study is needed of the modification and use in children.

### bCPAP efficacy scoring

As previously noted, higher cost bCPAP setups are widely used in high resource settings with good results (44). Hospitals in resource constrained settings often use “home-made” bCPAP setups with varying results (45, 46). To ensure that these are safe and effective, a simple scale has been developed based on the fluid mechanics of constant positive pressure delivery (47). If the expiratory limb is clean, wide (≥10 mm) and continuously bubbling, there is a similar pressure at the bubbling air/water interface and in the respiratory circuit proximal to the patient interface. If nasal prongs are occlusive of the nares, this pressure is adequately transmitted to the patient's nasopharynx. If bubbling is auscultated in both lungs, the pressure has been transmitted to both lungs. As a quick spot check, the clinician can also measure the pressures proximal to the prongs to ensure that the patient is receiving the prescribed CPAP treatment. Taken together, all these components can be utilized to calculate a score to assess the effectiveness of delivery of bCPAP. A score of 0–5 indicates ineffective bCPAP, 6–9 inconsistent bCPAP and a score of 10 suggests likely effective bCPAP (Table 2).

**Table 2:** The BCPAP score for effectiveness of bubble CPAP delivery.

	0	1	2	Total
Bubbles	Not present	Intermittent	Continuous	
Circuit	Contaminated (e.g. mold, biofilm, dirt)	Clean but small diameter (<10 mm)	Clean and wide diameter (≥10 mm)	
Prongs	Too small	Too large	Occlusive fit	
Airway	Blocked	Partially blocked	Open with bilateral breath sounds	
Pressure	Air leak	Intermittent	Maintained at set level	

Table re-used with permission from Oxford Press. “Contaminated” refers to the presence of mold, biofilm, dirt, etc.

By regularly using a stepwise approach from the bubbler through the circuit and nasal interface to the patient's lungs, clinicians can ensure safe and effective delivery of positive airway pressure therapy. Deviating from this protocol to use of narrow bore tubing in the expiratory limb should be approached with caution as this could result in the delivery of higher pressures depending on the air flow rate and the degree of occlusion at the nasal prongs (48). Because of the discordance between increased pressure seen in laboratory models of home-made bCPAP setups with a narrow bore expiratory limb (<10 mm) and the very few documented clinical concerns with these low-cost versions of bCPAP the authors are conducting a study to further investigate actual clinical problems seen with various forms of CPAP used in LMICs.

### Low-cost oxygen blender

Low-cost, constructible oxygen blenders are another example of a bCPAP modification which increase the feasibility of implementing these devices in LRS (49). As described above, bCPAP is an invaluable tool for treating pediatric respiratory disease in LRS. However, it is very common in these settings for low-cost bCPAP to be powered by pressurized 100% oxygen from a tank. As multiple studies have demonstrated, providing 100% oxygen introduces the risk of hyperoxia, and thus has deleterious effects on all ages, particularly neonates and critically ill children (50–52). In HICs, oxygen blenders are commonly employed to titrate oxygen concentrations between 21% and 100%, but these devices often cost several hundred U.S. dollars per unit, making them cost prohibitive for many LRS.

In the same way bCPAP is inexpensive and constructible, a novel oxygen blender that can be readily made using two 3 ml syringes with rubber stoppers, a 22G hypodermic needle, oxygen supply tubing, a nasal cannula circuit, tape, and a sharp cutting edge (scalpel or razor blade are preferred) has been designed and tested in the laboratory (49). The blender is currently being evaluated for use in children. The blender is positioned in-line with the bCPAP circuit between the oxygen source and the patient (Figure 1B). The mechanism utilizes the Venturi effect to entrain ambient air (21% oxygen) into the 100% oxygen stream, diluting the oxygen concentration to less than 100%. Similar principles have been utilized for other low-cost blender designs prototyped with 3D printing (53).

Preliminary studies have shown that use of this device can be taught to new English-speaking users in the span of about an hour and can be constructed in about 15 min once proficient (49). The blender can be built using one of two different sized entrainment ports which allows for two options of oxygen concentrations: 40%–50% or 60%–70%. The device also has a built-in safety check whereby the bubbling decreases when if the entrainment port is covered, often with a finger, which indicates that the device is working properly.

There are many advantages to this design. This is a cost-effective intervention, as the materials required for assembly total approximately $5USD. Furthermore, access to these materials is commonplace, even in the most resource-restricted hospitals. Therefore, spare parts are readily available in most healthcare settings. We have found that workshops including live demonstration, video, and guided instruction over the course of 1–2 h are sufficient to begin use. Monitoring of the device function is easily seen by the presence or absence of bubbling in the water bottle, as loss of flow or entrainment usually results in loss of bubbling. Lastly, the blender is powered by compressed oxygen and therefore does not rely on a stable source of electricity or medical air to function.

While there are many benefits to this blender, there are some notable limitations. Because of the design of the blender, it is not easily cleaned and therefore intended to be a single patient-use device, which contributes to increased medical waste. However, as many respiratory support circuits are designed to be single use, this is not a unique phenomenon. Another potential downside is that the syringe chamber is operating under high pressure and therefore prone to fragmenting if used with higher oxygen flows, which limits its usability to CPAP levels of 10 cm H2O or less and flow rates of 6 liters per minute or less. Fortunately, the entrainment of air allows for appropriate flows that reach the patient, however attempting to provide higher levels of CPAP remains a limitation of the blender circuit.

Taken together, the blender is an extremely low-cost method of delivering CPAP and appropriate levels of oxygen therapy when the only alternatives may be CPAP with 100% oxygen or no CPAP at all. While ideally all hospitals treating children with respiratory distress should have bCPAP machines with titratable oxygen levels available, not all do, thereby creating an environment where this low-cost blender can prove useful. A clinical trial to test its feasibility and safety in a LRS is currently underway.

### NeoVent—bubble NIPPV

While bCPAP is effective for infants in mild to moderate respiratory distress, infants with worsening or more severe respiratory failure can benefit from additional support such as Nasal Intermittent Positive Pressure Ventilation (NIPPV). NIPPV consists of a constant baseline pressure with intermittent positive pressure ventilation to reduce the patient's work of breathing (54). The clinician can set a peak inspiratory pressure (PIP) above a set positive end expiratory pressure (PEEP) with a cycling rate to additionally support oxygenation and ventilation. NIPPV has been used to prevent intubation and decrease post extubation failure, with particular benefit in premature infants or those with apnea (55, 56). Conventionally, in HRS, NIPPV has been delivered using expensive, complex electric ventilators in a noninvasive ventilation mode. In many LRS, NIPPV is often delivered with manual bag mask ventilation, which is only sustainable for a few hours.

**Figure 2:**
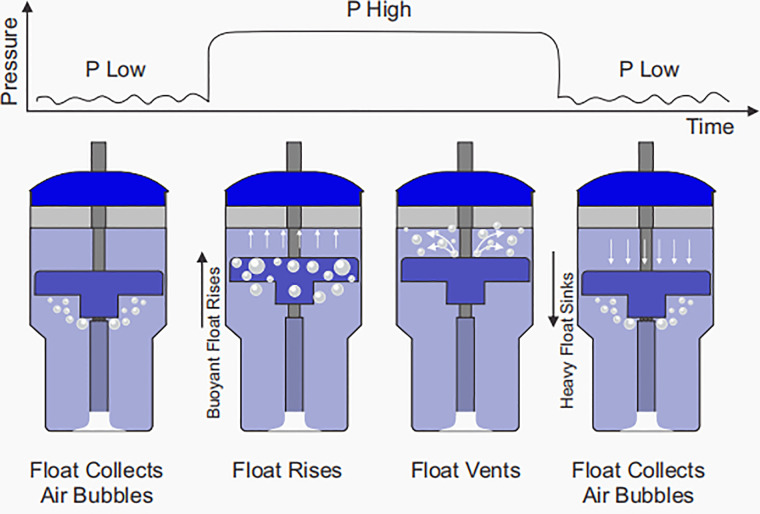
the NeoVent Bubble-NIPPV device. Figure used with permission from AIM Tech.

A novel bubble NIPPV device (NeoVent) has been developed which attempts to preserve the simple, non-electric design of bCPAP while providing additional support for infants in respiratory distress (57, 58). As previously described, with bCPAP, the delivered pressure is set hydrostatically by the submerged depth of bubbling. By altering the submerged depth of bubbling between two levels (e.g., 5 cm H2O and 20 cm H2O), bubble NIPPV can be delivered. This is achieved with a variable buoyancy float. Bubbles emerge from the submerged expiratory limb and the low pressure is delivered. The float collects these bubbles, becomes buoyant and rises. In the process, the float moves an attached sleeve which occludes the bubbling holes of the expiratory limb, so that a high pressure is delivered. The float then vents the air, becomes heavy and falls, reopening the bubbling holes and causing the pressure to return to the lower level. This process cyclically repeats, affecting a dual pressure waveform (Figure 2). The device has been designed to limit the time at *P* high to approximately one half of a second to prevent breath stacking. As in the case of bCPAP, bubble NIPPV is non-electric with a few components that can easily be set up, monitored, taken apart and cleaned. The delivered pressures and volumes have been optimized for supporting infants. Physicians, nurses, and family members can easily assess the device's function: when the float is “up”, pressure is “up” and when the float is “down”, pressure is “down”. As with bCPAP, if there is a significant leak, the bubbling will cease. The technology is currently undergoing clinical studies of safety.

## Implementation of new devices and techniques

When developing any new device or treatment it is essential to work together in a true partnership with a multidisciplinary team in the LMIC including clinicians, biomedical engineers, and technicians, allowing them to describe to the HIC partners what they actually need. The team should work together to determine the needs and ensure that on the ground providers understand how the innovation actually works. This collaboration will help with buy-in from the local providers, increasing its use and ultimately building the capacity for that facility to care for sicker patients, decreasing the need to transfer patients to higher-level facilities, and all the costs that are associated with that process. It is also important to recognize the challenges of conducting research in settings where research is not routinely conducted. It may be quite difficult to get staff buy-in about new studies when they are already very busy with patient care responsibilities. An on-the-ground champion for the study who is knowledgeable about the study protocol as well as the device being tested is vital for success, however this places substantial pressure on that individual. These responsibilities are best served by a dedicated research team whenever feasible.

As we move many of these treatments and devices from research into clinical practice, the implementation process is essential for ensuring that these devices are utilized as a means of decreasing morbidity and mortality. Challenges already being addressed by programs such as NEST-360 (59) and others include implementing proven therapies like bCPAP for neonates into the lower acuity health centers responsible for delivering, stabilizing and then referring sick neonates to higher levels of care.

Successful implementation of any technology requires trained biomedical engineers and/or technicians in addition to trained clinicians. Multiple sources including the WHO (60) highlight the “equipment graveyards” which litter hospital and healthcare facilities in LRS. These are filled with donated equipment and supplies that came broken or need a voltage step down, had missing critical parts or single use essential pieces not available in the LMIC, and equipment that was never appropriate for the specific facility to which it was donated because of the level of care they were able to provide (i.e., ventilator with no source of piped gas in the facility). Similarly, it is often seen that equipment that worked well for a time but then later malfunctioned is unable to be repaired due to lack of a specialist with the appropriate skills, and the equipment is ultimately discarded. Appropriately trained local biomedical engineers, technicians and their teams can be empowered to maintain and repair life-saving equipment vital to their healthcare facility. The appropriate maintenance of equipment is also facilitated by having service agreements coupled with the purchase of each piece of equipment. This helps hold the companies responsible for supporting high quality, durable equipment as well as recycling all equipment that can no longer be used or repaired.

Implementation and sustainability also require training of the entire healthcare team, including not just physicians but also nurses, respiratory therapists, and everyone responsible for caring for infants and children. This training must be appropriate for the given tasks of each team member and must include an ongoing plan of refresher courses. Examples of successful training packages include the Helping Babies Survive program which is now being rolled into the WHO's Essential Newborn Care package (61), the Fundamentals of Critical Care from the Society of Critical Care Medicine (with an adapted version for adults in LRS) (62).

Sometimes the best option for training is retraining existing staff for another task, such as training a nurse working in a pediatric or neonatal intensive care unit to function as a respiratory therapist in the same unit. While this can be an effective option that leverages existing resources, ensuring that retraining will lead to long-term reassignment is essential. Working with administrators so that trained staff are not constantly moved to other units shortly after they are appropriately trained for a given unit is critical. To stop this practice, administrators must be engaged and supportive, ensuring that they understand why it is important to develop staff and allow them to remain in their newly assigned units to enable successful implementation. Training works best when it is stepwise and building on previous skill sets and trainings. For example, it is often useful to first provide training on the basics of intensive care nursing and respiratory therapy such as the use of low-flow nasal cannulas, suctioning techniques, setting up and responding to monitors. Thereafter training can be advanced to non-invasive respiratory support, recording vital signs and using critical care flowsheets, with subsequent introduction to invasive ventilation only when both personnel and infrastructure support for using it safely and effectively is established. This stepwise and thoughtful progression allows the development of high-quality special care units.

## Future directions for research and development

Looking to the future, there remains a significant need for development, adaptation, and refinement of advanced respiratory support equipment for resource constrained settings. Areas of ongoing need include further development of ways to blend, warm, and humidify oxygen and air, low-cost but durable battery (or solar) powered oxygen concentrators, low-cost ventilators and video laryngoscopes for facilities that can support such technology, and reusable laryngeal-mask airways of appropriate sizes. Sustainable sources of items that are essential but cannot be reused, such as testing strips, or cartridges for point of care machines, are also necessary. Teams should also be encouraged to develop low-cost monitoring devices including multi-mode monitors with as many reusable pieces as possible. Additional needs include laboratory investigations such as point-of-care blood gas machines, durable reusable end-tidal carbon dioxide monitors and micro-sample tubes.

Countries should be prompted to include support medications such as vasopressors, sedation, pain medications, caffeine citrate and surfactant in their essential medication lists. Sustainability and appropriate local adaptations would be improved greatly if local entrepreneurs and companies were encouraged to participate using locally available resources to develop and manufacture these supplies and equipment. Biomedical engineers must be brought into the team, and subsequently empowered, trained, and given an enabling environment to be creative and innovative. There are many examples of this being successfully done in countries such as India (63) but far fewer examples in sub-Saharan Africa where it is often difficult for to import critical supplies and equipment. The more each country can be encouraged to develop their own high quality durable products, equipment, and resources, the more sustainable these products and processes become.

LMICs must also be supported in their efforts to develop, sustain, and improve local training programs to ensure that their healthcare providers are equipped to provide excellent care in their LRS. Whenever possible, high-income partners should support local training to help slow down the ongoing depletion of vital healthcare staff who often leave their home countries in pursuit of opportunities in higher resourced settings—the so-called “brain drain”. Efforts should be made to provide adequate remuneration, training and retraining as well as access to supplies and equipment needed to provide excellent care in an environment where well-trained local providers are encouraged, supported, and valued to encourage local staff retention. When striving for excellence, there should be collaboration between HIC provider teams and LMIC teams to share best practices and lessons learned as may be applicable and acceptable to that locale.

When providing increasingly more complex and intensive care, all team members must factor in the cultural, environmental, religious, and social implications of the suggested care. This will include weighing in practical points such as “who will take care of the other children when a neonate is hospitalized for months”; “how many other children in the family will miss school this year because their sibling in the pediatric intensive care used all the family funding for school fees”; and “what are the long-term implications of keeping a neonate or child separated from their family for extended periods of time or on the siblings when the parents are unavailable to meet the needs of the whole family while providing skin-to-skin care and breast milk to their extremely premature neonate”.

Lastly, provision of intensive care is globally expensive, in many settings in LMICs healthcare costs are paid out of pocket and thus unaffordable. Governments at every level have the power to invest in health insurance systems especially for the most vulnerable populations.

## Conclusion

Children living in LMICs continue to suffer from the highest proportion of childhood mortality in the world. Most of this burden is associated with respiratory diseases. Therefore, increasing access to therapies that are specifically targeted for these diseases and are adapted for this population are vital to reducing this mortality burden. Put another way, an ideal therapy or product should not only be effective in supporting the child's physiology, but also be affordable; easy to monitor, setup, clean, and repair; not rely on unstable energy sources (i.e., continuous electricity); and be durable. As above, SEAL-bCPAP, the syringe oxygen blender, and NeoVent bubble NIPPV are all examples of innovations that have taken these characteristics into consideration and have been successfully implemented to various degrees. However, more work remains to be done in all sectors of this work, including needs assessment, conceptualization, development, implementation, and maintenance. Altogether, this requires a collaborative effort between interprofessional HIC and LMIC teams to generate and disseminate the tools necessary to decrease the burden of respiratory diseases worldwide. Ultimately, we invite a global, unified, and collaborative effort to invest in the development of more accessible tools, technologies, and techniques such as those highlighted here.
